# Environmental cold exposure and cardiovascular mortality: biological mechanisms, regional heterogeneity, and public health implications

**DOI:** 10.3389/fpubh.2026.1961371

**Published:** 2026-09-30

**Authors:** Ling-Jie Xiao, Xin-Yu Ma, Ji-Zu Shi

**Affiliations:** 1 College of Sports Training, Jilin Sport University, Changchun, Jilin, China; 2 College of Physical Education, Northeast Electric Power University, Jilin, Jilin, China

**Keywords:** cardiovascular mortality, climate adaptation, cold exposure, environmental health, regional heterogeneity, social determinants of health

## Abstract

**Background:**

Ambient cold exposure has been associated with increased cardiovascular mortality worldwide, but effects vary across climatic and socioeconomic settings. This narrative review synthesizes epidemiological evidence on geographic variation in cold-related cardiovascular mortality and considers environmental, biological, and societal factors that may contribute to the observed patterns.

**Methods:**

We conducted a narrative review of epidemiological studies investigating associations between ambient cold exposure and cardiovascular mortality. Climatic setting and socioeconomic context were considered as separate analytical dimensions, using local temperature distributions, seasonal patterns, study-specific cold definitions, income classification, housing and heating conditions, healthcare accessibility, and other contextual characteristics where relevant. Because study designs, exposure definitions, lag structures, outcomes, and effect measures were heterogeneous, the synthesis was qualitative; no formal quantitative pooling, causal attribution, or direct ranking of regional vulnerability was undertaken.

**Results:**

Evidence suggests that cold-related cardiovascular mortality varies across climatic and socioeconomic settings. Among 26 epidemiological studies contributing to the regional synthesis, substantial heterogeneity was observed in study design, population coverage, temperature metrics, cold definitions, lag structures, covariate adjustment, analytical approaches, outcomes, and effect measures. Methodological credibility was considered qualitatively rather than through a formal risk-of-bias score, with greater interpretive weight given to large multi-location, nationwide, or within-country studies applying standardized or internally consistent analytical frameworks. Illustrative reported estimates included approximately 6.3% of cardiovascular deaths attributable to temperatures below the county-specific minimum mortality temperature in a US analysis, 11.4% (95% CI 6.0–15.4%; lag 0–14 days) attributable to temperatures below the city-specific optimum temperature in a seven-city Norwegian analysis, and a cold-wave-related cardiovascular mortality RR of 1.076 (95% CI 1.061–1.090; lag 0–3 days) in a nationwide Thai study. Because exposure definitions, lag structures, analytical approaches, outcomes, and effect measures differed substantially among studies, no consistent quantitative gradient in vulnerability by climatic setting, latitude, or socioeconomic context could be established. The qualitative evidence suggests that regional patterns may reflect a combination of climatic conditions, biological susceptibility, behavioral responses, socioeconomic circumstances, housing and heating conditions, healthcare capacity, population-level adaptive capacity, and methodological differences. Their relative contributions cannot be determined from the available evidence, and evidence from many warm-climate and lower-resource settings remains limited.

## 1 Introduction

Cardiovascular disease (CVD) remains a leading cause of death worldwide and imposes a substantial global public health burden (1). Traditional risk factors, including smoking, excessive alcohol consumption, hypertension, diabetes, and unhealthy lifestyles, remain major contributors to CVD; however, environmental exposures also influence cardiovascular health (2). Among these environmental determinants, ambient temperature is increasingly recognized as an important risk factor for cardiovascular morbidity and mortality (3, 4).

Experimental and physiological studies show that acute cold exposure can activate the sympathetic nervous system, induce peripheral vasoconstriction, elevate arterial blood pressure and myocardial oxygen demand, impair endothelial function, and promote hemoconcentration and prothrombotic responses (5–8). These findings provide biological and clinical plausibility for adverse cardiovascular effects of cold exposure, but they do not by themselves establish an effect on population-level cardiovascular mortality. Mortality evidence comes from population-based epidemiological studies and multi-country analyses that have reported higher cardiovascular mortality during low-temperature or extreme-cold conditions (9–11).

The public health burden attributable to cold exposure is substantial. In a US county-level analysis covering 2000–2020, an estimated 42,735 CVD deaths per year were attributable to temperatures below the county-specific minimum mortality temperature, corresponding to 6.3% of annual CVD deaths (12). In 15 Chinese megacities during 2007–2013, 15.8% (95% empirical CI 13.1–17.9%) of CVD deaths, corresponding to 305,902 deaths, were attributable to temperatures below city-specific minimum mortality temperatures (13). At the global level, Hundessa et al. estimated 1,801,513 annual CVD deaths associated with nonoptimal temperatures during 2000–2019; 1,666,814 were associated with cold temperatures (14). A separate GBD 2019 analysis estimated 1,104,200 low-temperature-attributable CVD deaths in 2019 (15, 16). These estimates were derived from different populations, periods, exposure definitions, modeling frameworks, and burden metrics and therefore should be interpreted as context-specific rather than directly comparable measures.

Associations between cold exposure and cardiovascular mortality have been reported across many climatic settings, but their magnitude varies substantially among populations (11, 17–21). Systematic reviews also show appreciable heterogeneity in cold-related cardiovascular and broader health effects (22). This variation may reflect differences in climate, population characteristics, socioeconomic and infrastructural conditions, exposure definitions, and analytical methods. Understanding these sources of heterogeneity is important for interpreting existing evidence and for developing regionally appropriate cardiovascular risk assessments.

However, the determinants of geographic variation in cold-related cardiovascular mortality remain incompletely understood. Existing epidemiological studies differ substantially in population characteristics, climatic settings, exposure metrics, analytical methods, and contextual factors. Moreover, population-based time-series and ecological studies have limited ability to separate the contributions of biological susceptibility, behavioral responses, socioeconomic conditions, housing and heating environments, and healthcare-related factors. An integrated synthesis is therefore needed to clarify both the evidence and its limitations.

In this review, climatic adaptation is not treated as a single measurable exposure or an established causal determinant. Rather, it is operationalized within the broader concept of population-level adaptive capacity, comprising three distinct but interacting dimensions: physiological acclimatization to repeated cold exposure, behavioral adaptation such as clothing, activity, and heating use, and social/infrastructural adaptation including housing, heating systems, energy access, warning systems, and healthcare capacity.

These dimensions are considered qualitatively according to how they were assessed or reported in the available studies, rather than being combined into a single quantitative adaptation index.

Previous reviews have examined cardiovascular responses to cold exposure and the broader relationships between climate, ambient temperature, and cardiovascular health (3, 4, 23). Building on this literature, the present review places particular emphasis on geographic heterogeneity in cold-related cardiovascular mortality and qualitatively integrates epidemiological evidence with biological, climatic, socioeconomic, infrastructural, healthcare-related, and adaptive factors that may contribute to variation across populations.

In this narrative review, we synthesize epidemiological evidence on regional heterogeneity in cold-related cardiovascular mortality from a global perspective. We examine regional patterns in relation to local climatic conditions and temperature characteristics and consider the adaptation dimensions defined above together with physiological susceptibility, socioeconomic conditions, housing and heating environments, and healthcare accessibility. We also summarize biological mechanisms and methodological factors that may influence cross-regional comparisons. Finally, we present an integrative conceptual framework for interpreting how environmental exposure, population adaptation, and societal resilience may interact in shaping regional variation in cold-related cardiovascular mortality.

## 2 Literature search strategy and evidence selection

This narrative review was conducted to synthesize epidemiological and mechanistic evidence on environmental cold exposure and cardiovascular mortality, with particular emphasis on regional heterogeneity and its potential biological, climatic, socioeconomic, infrastructural, and healthcare-related determinants. Methodological transparency and reporting were strengthened with reference to SANRA and established guidance for conducting and reporting narrative reviews (24–26).

A literature search was conducted in PubMed, Web of Science Core Collection, Embase, the Cochrane Library, and China National Knowledge Infrastructure (CNKI) from database inception to 30 June 2026. No lower publication-date limit was applied. The search strategies were rerun on 2 September 2026 to document the final database syntax and confirm that the reported queries remained executable; eligibility remained restricted to publications published on or before 30 June 2026. Because the original database-level retrieval counts were not preserved contemporaneously, counts from the later rerun were not used as a surrogate for the original screening denominator. The core Boolean structure combined terms for cold exposure with cardiovascular disease and mortality: (“cold exposure” OR “low temperature” OR “ambient temperature” OR “cold weather” OR “cold spell” OR “cold wave”) AND (“cardiovascular disease” OR “cardiovascular mortality” OR “cardiovascular death”). Complete database-specific strategies, including controlled vocabulary, free-text terms, field tags, truncation, Boolean operators, search dates, and database-specific syntax, are provided in Supplementary Appendix S1.

For consistency of terminology, several exposure terms are distinguished throughout this review. For the epidemiological synthesis and regional comparisons, cold exposure is used as an umbrella term referring to exposure to ambient or environmental temperatures considered cold relative to the reference used in the original study; it encompasses both continuous low-temperature exposure and discrete cold-spell or cold-wave events. Controlled laboratory or physiological exposures are identified separately as experimental cold exposure where relevant. Low temperature refers to temperature values below a study-specific reference, such as the minimum mortality temperature (MMT), theoretical minimum-risk exposure level (TMREL), a locally defined percentile, or an absolute temperature threshold, and therefore does not denote a single universal temperature across studies. Moderate cold refers to the non-extreme portion of the cold-related temperature range below the study-specific optimum or minimum-mortality temperature but above the threshold used to define extreme cold. Where studies partitioned temperature-related burden using percentile-based definitions, the original study-specific cut-points were retained rather than imposing a common percentile across studies. Cold spell or cold wave refers to an episodic exposure defined in the original study by temperatures below a specified absolute or percentile-based threshold for a specified number of consecutive days. Because both the temperature threshold and required duration vary substantially among studies, these event-based terms are used only according to the definitions reported by the original investigators and are not treated as equivalent to continuous low-temperature exposure. Accordingly, no universal percentile, absolute temperature, or minimum duration was imposed across the included studies.

Reference lists of eligible original studies and relevant systematic reviews and meta-analyses were additionally screened to identify potentially relevant publications not retrieved through the electronic searches.

The search was restricted to peer-reviewed publications in English or Chinese. Gray literature, including dissertations, conference proceedings without full peer-reviewed reports, preprints, government reports, and other unpublished materials, was not systematically searched. Publications in languages other than English or Chinese were not included. Study authors were not contacted to obtain unpublished results, additional analyses, or missing data.

For the principal epidemiological synthesis, studies were eligible if they met the following criteria: (1) human population-based epidemiological studies, including time-series, case-crossover, ecological, multicity, multi-location, or other observational designs reporting population-level mortality; (2) assessment of ambient or environmental cold exposure, including continuous low-temperature exposure or study-defined cold-spell/cold-wave events; (3) cold exposure defined using a study-specific reference such as the minimum mortality temperature, theoretical minimum-risk exposure level, percentile-based threshold, absolute temperature threshold, or duration-based cold-spell definition; (4) reporting cardiovascular mortality or cause-specific cardiovascular mortality as an outcome; (5) provision of information relevant to exposure-response relationships, attributable burden, geographic variation, susceptibility, or effect modification; and (6) publication in English or Chinese. No geographic restriction was applied. Multi-country, multi-location, and within-country studies using standardized or internally consistent analytical approaches were given greater interpretive weight for cross-regional comparison.

Studies were excluded from the principal epidemiological synthesis if they: (1) involved non-human populations; (2) assessed heat exposure only without reporting cold-specific results; (3) examined morbidity, hospitalization, physiological responses, or other intermediate outcomes without reporting cardiovascular mortality; (4) did not provide sufficient information to determine the relevant cold exposure or cardiovascular mortality outcome; (5) were commentaries, editorials, conference abstracts without adequate methodological or quantitative information, or duplicate reports of the same analysis; or (6) fell outside the predefined publication-language or date criteria. Where multiple publications analyzed overlapping populations, the report providing the most relevant or comprehensive information was preferentially retained, while complementary reports were included only when they contributed distinct outcomes or methodological information. Publications based on overlapping source datasets were retained only when they addressed distinct analytical questions or provided complementary outcomes; such publications were treated as overlapping analyses rather than as independent study populations.

Because the review also aimed to explain the biological plausibility and contextual determinants of regional heterogeneity, supporting evidence was considered using a broader framework. Experimental and physiological studies were used to inform biological mechanisms, whereas studies addressing housing, heating, socioeconomic circumstances, healthcare accessibility, behavioral responses, air pollution, respiratory infections, and other relevant modifiers were used to inform the interpretation of potential determinants. Systematic reviews and meta-analyses were used primarily to contextualize the evidence, identify additional primary studies, and assess consistency with the broader literature rather than being treated as independent regional effect estimates.

To avoid conflating evidence with different inferential roles, the included literature was interpreted within four distinct evidence domains. First, epidemiological studies reporting cardiovascular mortality or cause-specific cardiovascular mortality constituted the primary evidence base for statements regarding mortality associations, attributable burden, and regional heterogeneity. Second, studies reporting cardiovascular morbidity or intermediate clinical outcomes, including hospitalization, blood pressure, myocardial ischemia, vascular function, and related physiological responses, were used only to inform clinical plausibility and susceptibility and were not treated as direct evidence of cardiovascular mortality effects (27). Third, experimental and laboratory cold-exposure studies were used to characterize biological mechanisms and physiological responses to cold but were not used to estimate population-level mortality risk. Fourth, studies of housing, heating, socioeconomic conditions, healthcare access, and related policies were used to identify potential social, infrastructural, or policy modifiers; unless they directly evaluated mortality outcomes, they were not interpreted as demonstrating an effect on cardiovascular mortality. Accordingly, statements concerning cardiovascular mortality were based primarily on studies with mortality endpoints, whereas the other evidence domains were used for mechanistic or contextual interpretation.

Titles and abstracts retrieved through the searches were assessed for relevance, followed by full-text examination of potentially eligible publications. Literature selection and data extraction were performed by L-JX. For all 26 epidemiological studies included in the regional synthesis, extracted items included country or region, study period, study design, population characteristics, temperature metric, cold or cold-spell definition, exposure duration or lag, cardiovascular outcome, effect estimate, attributable mortality or burden, and reported modifying factors; these items are summarized in Table 1. The evidence-selection process was structured according to the predefined eligibility and thematic criteria described above, while retaining the narrative-review design rather than applying a formal systematic-review framework. Following relevance assessment and full-text examination, 84 publications were retained for the narrative evidence base. Among these, 18 informed the biological-mechanism section, 26 contributed to the regional epidemiological synthesis, and 42 informed the discussion of determinants, modifiers, and public health implications. Because individual publications could contribute to more than one thematic domain, these categories were not mutually exclusive. Three additional publications addressing review methodology and reporting were included in the reference list but were not counted as part of the substantive evidence base.

**Table 1:** Characteristics and principal findings of the 26 epidemiological studies included in the regional synthesis of cold exposure and cardiovascular mortality.

Study	Country/region	Study period	Study design	Population size/characteristics	Temperature metric	Cold / cold-spell definition	Exposure duration/lag	Cardiovascular outcome	Effect estimate/mortality increase	Attributable mortality/burden	Reported modifying factors
Al-Kindi et al. (15)	Global; analyses stratified by world region and sociodemographic index (SDI)	1990–2019	Standardized global comparative burden analysis (GBD 2019 methodology)	Global population represented in GBD 2019; CVD deaths and DALYs; analyses stratified by SDI and world region	Location-specific theoretical minimum-risk exposure level (TMREL), defined by the temperature associated with the lowest mortality rate	Low temperature below the location-specific TMREL	Not applicable; annual attributable-burden estimates and temporal trends	CVD deaths and disability-adjusted life years (DALYs)	Low-temperature-related CVD deaths increased by 36% (95% UI 25–48) from 1990 to 2019	In 2019, 1,104,200 (95% UI 897,783–1,326,965) CVD deaths and 19,768,986 (16,039,594–23,925,945) DALYs were attributable to low temperature	World region, SDI and income group
Alahmad et al. (11)	567 cities in 27 countries	1979–2019 (period varied by location)	Standardized multi-country time-series analysis using the Multi-Country Multi-City Collaborative Research Network protocol	More than 32 million cardiovascular deaths across 567 cities	Daily mean temperature; location-specific temperature distribution	Extreme cold defined as temperatures below the location-specific 2.5th percentile	DLNM with delayed effects; standardized analytical protocol	Overall CVD and cause-specific cardiovascular mortality	Extreme-cold days were associated with excess cardiovascular mortality; effects varied by cardiovascular cause and location	Approximately 9.1 excess CVD deaths per 1,000 cardiovascular deaths during extreme-cold days in the pooled analysis	Location, cardiovascular cause, and local temperature distribution
Eurowinter Group (21)	Eight European regions: north and south Finland, Baden-Württemberg, the Netherlands, London, north Italy, Athens and Palermo	1988–1992; Athens and Palermo data from 1992	Standardized multi-region ecological/time-series analysis using generalized linear modeling, with a parallel survey of cold-protection practices	Men and women aged 50–59 and 65–74 years; 24 age-sex-region groups in six regions with full data; death count NR	Daily outdoor temperature; percentage increase in daily mortality per 1°C decrease below 18 °C	Outdoor temperature below 18 °C; protective-factor survey results standardized to 7°C outdoor temperature	Daily exposure; lag period not separately reported in the abstract	Ischemic heart disease, cerebrovascular disease, respiratory disease, and all-cause mortality	Standardized slopes demonstrated cross-regional heterogeneity. The abstract’s numerical example was all-cause mortality: Athens 2.15% (95% CI 1.20–3.10) vs. south Finland 0.27% (0.15–0.40) per 1°C decrease	NR	Baseline mortality, age structure, sex and influenza were considered in cross-region comparisons. Winter climate, indoor temperature, bedroom heating, clothing and outdoor activity were examined; associations with these protective factors were significant for all-cause/respiratory mortality but not for IHD or cerebrovascular mortality (*p* > 0.05)
Salerno et al. (12)	United States	2000–2020	County-level analysis	819 counties; adults ≥25 years; ~81.9% of the 2010 US population; 14,180,068 CVD deaths	Monthly mean ambient temperature; county-specific MMT	Not applicable	Monthly exposure; lag 0	CVD mortality (ICD-10 I00–I99)	Nonlinear temperature–mortality association; no single summary RR reported; cold accounted for most temperature-related burden	Approximately 42,735 cold-attributable CVD deaths/year (95% empirical CI 40,717–44,956); 6.3% (95% empirical CI 6.0–6.6%) of annual CVD deaths	Geographic heterogeneity; individual sociodemographic effect modification not formally assessed
Vázquez Fernández et al. (47)	Norway	1996–2018	Time-series analysis	Daily mortality records from 7 Norwegian cities; total CVD death count NR	Daily ambient air temperature; city-specific optimum temperature	Not a cold-spell study; extreme cold defined as <1st percentile	DLNM; lag 0–14 days	CVD mortality	No single common effect estimate summarized here; cold-related mortality burden predominated over heat	11.4% (95% CI 6.0–15.4) of CVD deaths attributable to cold; extreme cold ~0.3%	Variation across cities, age and sex
Ryti et al. (67)	Oulu, Finland	1998–2011	Case-crossover study	3,614 autopsy-verified sudden cardiac deaths	Location-specific daily temperature at home address	≥3 consecutive days with daily minimum temperature below the season-specific 5th percentile	7-day hazard period preceding death	Sudden cardiac death	Cold spell: OR 1.33 (95% CI 1.00–1.78); each additional cold day: OR 1.19 (1.07–1.32)	NR	Season; cold-spell duration; ischemic vs. non-ischemic SCD
Yang et al. (13)	15 Chinese megacities	2007–2013	City-specific quasi-Poisson DLNMs followed by multivariate meta-analysis	1,936,116 CVD deaths	Daily mean temperature; city-specific MMT	Cold defined as temperatures below the city-specific MMT	Cumulative lag 0–21 days	CVD mortality	Temperature-attributable fraction 17.1% overall; most attributable burden was related to cold	Cold-attributable fraction 15.8% (95% empirical CI 13.1–17.9%), corresponding to 305,902 deaths	City, sex, age, and education level
Yang et al. (20)	South and West China	2008–2011	Multi-region time-series analysis using Poisson generalized additive models, followed by analysis of regional modifiers	26 Disease Surveillance Point regions; 103,218 CVD deaths	Daily mean temperature; low temperature referenced to region-specific 10th percentile	Not applicable	Cold effects assessed up to 14 days	CVD mortality (ICD-10 I00–I99)	Low temperature associated with increased CVD mortality in most regions, with substantial regional variation	NR	Education, age ≥65 years, sex; socioeconomic and demographic characteristics
Hu et al. (63)	Zhejiang Province, China	2009–2015	Two-stage county-level time-series analysis with DLNMs and meta-regression	89 counties; 54.4 million residents (22.2 million urban, 32.2 million rural); ~0.7 million CVD deaths	1-km gridded daily 24-h mean temperature; MMT reference	Not applicable	DLNM; lag 0–21 days	Cardiovascular and other cause-specific mortality	Cold-related mortality risks generally higher in rural than urban counties	Attributable fractions and deaths calculated separately for cold/heat; rural burden higher	Urbanization, age structure, education, GDP, healthcare services, air-conditioning, occupation
Lei et al. (87)	China (nationwide)	Cool seasons, 2013–2015	Nationwide multicity study	272 cities; nationally representative Disease Surveillance Points System	Daily mean temperature; cold defined using the 2.5th percentile	12 definitions; representative definition: ≤5th percentile for ≥4 consecutive days	Cumulative lag 0–28 days	CHD, stroke, non-accidental and COPD mortality	Under the representative definition: CHD RR 1.66 (95% CI 1.20–2.31); stroke RR 1.49 (1.12–1.97)	Nationwide cold-spell mortality burden estimated; magnitude varied by definition	Climatic region; cold-spell threshold and duration
Yang et al. (64)	Anhui Province, China	2016–2020	Province-wide time-series analysis comparing urban and rural areas	Province-wide daily MI deaths; urban and rural areas; total MI death count NR	Daily mean temperature; cold defined using the 2.5th percentile	Not a duration-based cold-spell study; percentile-defined cold exposure	Lag 0 reported for principal urban–rural comparison	Myocardial infarction mortality	Rural RR 1.13 (95% CI 1.02–1.26); urban RR 0.99 (0.80–1.21)	NR	Urban–rural residence
Ferreira et al. (52)	Brazil (6 metropolitan micro-regions)	1996–2013	Ecological time-series study	6 metropolitan micro-regions, 64 municipalities; midpoint population ~35.48 million	Daily mean ambient temperature	Not applicable	DLNM; lag up to 14 days	Acute myocardial infarction mortality	Cold-related risk varied markedly among regions; little association in regions nearest the equator	Cold-attributable deaths ranged from ~176/year in Brasília to ~661/year in São Paulo	Geographic and climatic region
Denpetkul and Phosri (53)	Thailand (65 provinces)	2010–2017	Two-stage time-stratified case-crossover analysis with multivariate meta-regression	65 provinces; 2,891,407 total deaths, including 403,450 CVD deaths	Daily ambient temperature; MMT as optimum	Not applicable	Cumulative lag 0–7 days	CVD mortality	V-shaped temperature–CVD mortality association; no single summary RR reported here	2.92% (95% empirical CI 0.55–5.10) of CVD mortality attributable to non-optimum temperature overall	Greenness (NDVI), especially for heat-related effects
Khunthong et al. (61)	Thailand (6 regions)	2014–2022	Nationwide time-series study	Daily CVD and respiratory mortality across 6 regions; data linked to 67 monitoring stations; total CVD death count NR	Station-specific temperature percentiles	9 cold-wave definitions using percentile thresholds over 2–4 consecutive days	Lag 0–21 days; principal pooled CVD estimate at lag 0–3	CVD mortality, including cerebrovascular mortality	Cold waves: RR 1.076 (95% CI 1.061–1.090), corresponding to ~7.6% higher CVD mortality	NR	Region, sex, age and cardiovascular subtype; females showed higher risk than males
Atsumi et al. (10)	Ibaraki Prefecture, Japan	1993–2008	Time-stratified case-crossover analysis within the Ibaraki Prefectural Health Study	3,593 subjects who died from CVD; mean cohort follow-up 9.7 ± 4.0 years	Daily maximum ambient temperature	Continuous temperature decrease; no cold-spell threshold	Temperature averaged over lag 0–2 days	All CVD and stroke mortality	Per 1°C decrease: all CVD OR 1.018 (95% CI 1.003–1.034); stroke OR 1.025 (1.003–1.048)	NR	Age <80 years and hyperglycemia; obesity, smoking, alcohol use and hypertension were also examined
Hundessa et al. (14)	Global; 1,847 locations in 32 countries/territories	Epidemiological data 1969–2019; burden trends 2000–2019	Three-stage global modeling study: location-specific quasi-Poisson DLNMs, multivariate meta-regression and grid-level burden estimation	Global grids covering ~6.9 billion people (98.5% of the global population)	Daily mean temperature; grid-specific MMT	Cold below grid-specific MMT; extreme cold ≤2.5th percentile	DLNM; cumulative lag 0–21 days	CVD mortality (ICD-9390–459; ICD-10 I00–I99)	Cold accounted for 8.20% (95% eCI 6.74–11.57%) of global CVD deaths, or 24.17 (19.86–34.10) deaths/100,000	~1,666,814 (95% eCI 1,369,292–2,351,730) cold-related CVD deaths annually	Annual mean temperature, temperature range, climate zone, continent, GDP per capita and time
Li et al. (16)	Global; 204 countries and territories	1990–2019	GBD 2019 comparative-burden analysis with age-period-cohort modeling	Global population represented in GBD 2019; CVD deaths and DALYs by country, region, sex and age	GBD low-temperature exposure relative to location-specific minimum-risk temperature	Low temperature below the location-specific minimum-risk temperature	Not applicable; annual burden and age-period-cohort trends	CVD, ischemic heart disease, hypertensive heart disease and stroke deaths/DALYs	Attributable CVD deaths increased from ~812,000 to ~1,104,000 (35.9%); age-standardized rates generally declined except in East Asia and the Pacific	~1.104 million low-temperature-attributable CVD deaths in 2019	SDI, sex, age, geographic region, CVD subtype, period and birth cohort
Gyllerup et al. (17)	Sweden (259 municipalities)	1975–1984	Municipality-level ecological multiple-regression analysis	Men aged 40–64 years in 259 municipalities; serum-cholesterol subsample of 37 municipalities; death count NR	Municipality-level cold index	Continuous regional cold-climate index; no event threshold	Ten-year ecological exposure; no short-term lag	Acute myocardial infarction mortality	Cold index was the strongest single explanatory factor and substantially increased model R^2^ after other risk factors	NR	Serum cholesterol, water hardness, socioeconomic factors, tobacco, butter and antihypertensive sales
Eng and Mercer (18)	Dublin, Ireland and Oslo/Akershus, Norway	1985–1994	Comparative ecological time-series and linear-regression analysis	Men and women aged ≥60 years; registered daily mortality for three CVD groups; death count NR	Daily mean air temperature and wind-chill temperature equivalent derived from wind speed	Continuous low temperature/wind-chill exposure; no threshold	Daily exposure; lag not separately reported	Mortality from three cardiovascular disease groups	Mortality was inversely correlated with air temperature and wind chill in all CVD groups; air-temperature slopes were steeper in Dublin	NR	Location, wind exposure, sex and age
Wu et al. (19)	Taiwan (358 townships)	1994–2003	Island-wide ecological spatial-regression analysis with principal-component analysis	358 townships; population-based CVD mortality; total death count NR	Interpolated township-level daily maximum, mean and minimum temperature	Cold event: maximum temperature decrease ≥8°C within 24 h or Taipei minimum temperature <10 °C; 24 events	Fourteen-day windows before and after each event	CVD mortality (ICD-9390–459)	Mean CVD mortality increased from 7.64 before to 8.29 per 100,000 after cold events (*p* < 0.001)	NR	Urbanization, medical resources, older/disabled/older-living-alone population, Aboriginal population and economic opportunity
Auliciems and Frost (46)	Montreal, Canada	1983–1984	Ecological time-series analysis	Daily myocardial infarction deaths over 2 years; death count NR	Daily temperature departures from seasonally determined thermally neutral conditions; snowfall	Cold-side deviation from seasonal thermal neutrality; anomalous cold spells examined	Daily exposure; previous-day snowfall; temperature lag NR	Myocardial infarction mortality	Mortality increased with departures below thermal neutrality; death frequency decreased during anomalous cold spells, interpreted as cold avoidance	NR	Season, snowfall and behavioral cold avoidance
Hajat et al. (51)	England and Wales (10 regions)	1993–2003	Region-specific ecological time-series Poisson models with pooled meta-regression	All registered deaths; CVD accounted for 41.0%; total death count NR	Daily mean temperature (mean of daily maximum and minimum)	Below the region-specific 5th percentile of mean temperature	Temperature averaged across cumulative lag 0–13 days	All-cause and cause-specific mortality, including CVD	Cold exposure increased CVD mortality in all regions; strongest regional cold effects were observed in eastern England; no single pooled CVD RR reported	NR	Age, care/nursing-home residence, urban–rural setting and deprivation; rural cold effects were slightly stronger in more deprived areas
Fu et al. (54)	India (nationally representative)	2001–2013	Case-crossover study with DLNMs and pooled climate-region estimates	411,613 medical deaths; 19,753 stroke and 40,003 IHD deaths among adults aged 30–69 years	1° × 1° gridded daily mean temperature; cause-specific MMT	Extremely cold: 0.4–13.8°C (≤2.5th percentile); moderately cold: 13.8 °C to cause-specific MMT	Cumulative lag 0–21 days; cold effects persisted for ~14 days	Stroke and ischemic heart disease mortality	Moderately cold attributable fraction: stroke 27.2% (95% eCI 11.4–40.2%); IHD 9.7% (3.7–15.3%)	In 2015, the CVD-specific total was NR; approximately 197,000 stroke, IHD, and respiratory deaths combined among adults aged 30–69 years were attributable to moderate cold.	Climate region, age, cause of death and previous vascular disease
Guo et al. (60)	Chiang Mai, Thailand	1999–2008	Quasi-Poisson time-series analysis with DLNM	Population ~1.60 million in 2008; 129,254 non-external deaths, including 11,746 CVD deaths	Daily mean ambient temperature	Cold comparison: 19.35°C (1st percentile) vs. 24.7°C (25th percentile)	Cumulative lag 0–21 days	CVD mortality (ICD-10 I00–I99)	Cold temperature significantly increased CVD mortality; effects were immediate and lasted longer than heat; no single CVD RR reported in text	NR	Mortality cause and age group (age stratification for non-external mortality); PM10, ozone and humidity were adjusted
Von Klot et al. (78)	United States (48 cities)	1992–2000	Multicity quasi-Poisson time-series analysis with random-effects pooling	All-age daily cardiac deaths; city means ranged from 1.4 to 142 deaths/day; total count NR	Daily mean temperature	Cold comparisons of 0 to −5°C and city-specific 10th to 1st percentiles	Lag 0 primary; longer lag averages examined in sensitivity analyses	Cardiac mortality (ICD-9390–429; ICD-10 I01–I51)	A decrease from 0 to −5°C increased cardiac mortality risk by 1.6% (95% CI 1.1–2.1%)	NR	City/climate and influenza activity; cold effect persisted after adjustment for influenza, humidity and air pressure
Meng et al. (79)	Shenzhen, China	2013–2017	Quasi-Poisson time-series analysis with DLNM comparing eight cold-spell definitions	65,325 all-cause deaths, including 23,030 CVD deaths	Daily mean ambient temperature	Optimal CVD definition: ≤3rd percentile for ≥4 consecutive days	Cumulative lag 0–14 days; significant effect began at lag 4 and lasted ~6 days	CVD mortality	Cold spell RR at lag 0–14: 1.58 (95% CI 1.00–2.48)	Attributable fraction 1.37% (95% eCI 0.05–2.51%); 142 CVD deaths	Sex and age; risks were higher in females and adults aged ≥65 years

Table 1 includes all 26 epidemiological studies that met the predefined eligibility criteria for the principal regional mortality synthesis. No eligible principal epidemiological study was omitted from the table. Systematic reviews and meta-analyses, experimental or mechanistic studies, studies reporting morbidity or intermediate outcomes only, studies reporting all-cause or non-accidental mortality without a cardiovascular-specific outcome, and contextual studies were not counted as principal epidemiological studies and are discussed separately according to their inferential roles. Effect estimates are presented according to the original exposure definitions and analytical frameworks of individual studies. Risk estimates per unit decrease in temperature, cold-spell-related effect estimates, attributable fractions, and excess-death estimates represent distinct epidemiological measures and should not be interpreted as directly comparable measures of effect magnitude. Cross-regional inference was based preferentially on studies applying standardized or internally consistent analytical frameworks across multiple locations; estimates from independent studies are presented for descriptive purposes only. Not applicable = the item does not apply to that study design; NR = not reported or not separately reported in the information available for that study. MMT, minimum mortality temperature; CVD, cardiovascular disease; CHD, coronary heart disease; MI, myocardial infarction; SCD, sudden cardiac death; DLNM, distributed lag nonlinear model; RR, relative risk; OR, odds ratio; NDVI, normalized difference vegetation index. GBD, Global Burden of Disease; TMREL, theoretical minimum-risk exposure level; SDI, sociodemographic index; DALY, disability-adjusted life year; UI, uncertainty interval; IHD, ischemic heart disease.

The narrative synthesis was organized using a structured thematic approach rather than formal quantitative aggregation. Epidemiological mortality evidence was first grouped according to climatic setting and geographic context, while socioeconomic characteristics were considered separately as contextual dimensions. For all 26 epidemiological studies included in the regional synthesis, study characteristics and effect estimates were tabulated by country or region, study period, temperature metric, cold or cold-spell definition, exposure duration or lag structure, cardiovascular mortality outcome, effect measure, attributable burden, and reported modifying factors (Table 1). Within each thematic section, findings were compared qualitatively with attention to the consistency of direction of association, differences in exposure definitions and analytical methods, and the comparability of effect measures. Mechanistic evidence was synthesized according to major biological pathways, whereas evidence concerning potential determinants of regional heterogeneity was organized into physiological adaptation, behavioral adaptation, housing and heating conditions, socioeconomic circumstances, healthcare resources, individual susceptibility, and environmental co-exposures. No formal thematic coding software or quantitative scoring system was used.

No formal study-level quality-assessment or risk-of-bias tool was applied to the included studies. Because the evidence base comprised heterogeneous study designs, including time-series, case-crossover, ecological, multicity, global comparative, experimental, and review evidence, no single risk-of-bias instrument was considered appropriate across all included publications. Instead, the methodological credibility and interpretability of individual studies were considered qualitatively according to study design, population coverage and sample size, exposure assessment, outcome ascertainment, control of major confounders, lag specification, statistical approach, and the comparability of exposure and effect measures. Greater interpretive weight was given to large multi-country, multi-location, or within-country studies applying common analytical frameworks across locations. For the interpretation of potential modifiers, greater weight was also given to studies that directly evaluated effect modification, stratified associations, within-country contrasts, or policy-related differences in relation to factors such as socioeconomic characteristics, urban–rural residence, housing, indoor thermal conditions, or heating availability, rather than to ecological correlations alone.

Differences in exposure assessment, including mean, minimum, or apparent temperature, study-specific cold thresholds, cold-spell definitions, exposure duration, and lag structure, were explicitly considered during the qualitative synthesis. Studies using markedly different exposure metrics, cold definitions, or lag structures were not pooled or directly compared on a common numerical scale. No formal numerical weighting or down-weighting scheme was applied; instead, such studies were treated as context-specific evidence and were given less interpretive weight for direct cross-regional comparisons. Greater weight was placed on multi-location or within-country studies that applied standardized or internally consistent exposure definitions and analytical frameworks across locations. This structured qualitative appraisal was used to guide interpretation and should not be interpreted as a formal study-level risk-of-bias assessment.

Because of substantial heterogeneity in study populations, climatic settings, exposure metrics, cold-spell definitions, lag structures, outcomes, and effect measures, formal quantitative pooling was not considered appropriate for the purpose of this narrative review. Risk changes per unit decrease in temperature, cold-spell-related relative risks, attributable fractions, and absolute excess-death estimates were therefore interpreted according to their original analytical contexts and were not numerically compared as equivalent measures of vulnerability. The main cross-regional conclusions were based preferentially on standardized multi-location analyses and internally comparable evidence, supplemented by eligible region-specific epidemiological studies where appropriate.

A completed SANRA-based self-assessment is provided in Supplementary Table S2 to document the reporting and methodological features of the narrative review.

## 3 Biological mechanisms linking cold exposure to cardiovascular mortality

### 3.1 Neuroendocrine activation

Activation of the sympathetic nervous system is among the earliest physiological responses to cold exposure (28, 29). Acute cooling stimulates sympathetic outflow, peripheral vasoconstriction, and changes in systemic vascular resistance and cardiac output (29, 30). These responses help conserve body heat but can also increase arterial pressure, myocardial oxygen demand, and cardiac afterload.

Cold-pressor experiments demonstrate acute increases in sympathetic nerve activity and blood pressure during intense localized cold stimulation (31, 32). However, the cold-pressor test is a short-duration experimental stressor and does not reproduce the intensity, duration, thermal distribution, clothing conditions, or behavioral adaptation that characterize ordinary community exposure to winter temperatures. These experiments therefore demonstrate autonomic responsiveness to acute cold stress rather than the magnitude of physiological responses during real-world ambient cold exposure. Sex-related differences in autonomic and neurovascular responses have also been reported (33), supporting possible variation in individual physiological susceptibility.

The cardiovascular consequences of sympathetic activation depend partly on baseline cardiovascular health. Healthy individuals generally maintain hemodynamic stability through intact autonomic regulation and adequate cardiovascular reserve. In people with established cardiovascular disease, cold-induced increases in vascular resistance and cardiac workload may be less well tolerated (5, 6). Clinical experimental evidence also indicates that extreme cold can lower the ischemic threshold in patients with coronary artery disease (34). These findings support clinical plausibility, but they should not be interpreted as direct evidence that a specific physiological pathway causes population-level cold-related mortality.

Taken together, these findings identify sympathetic activation as an important early component of the cardiovascular response to cold (5, 28). Autonomic activation alone, however, is unlikely to explain the observed population-level mortality association; downstream vascular, inflammatory, and hematologic responses may also contribute (35).

### 3.2 Vascular dysfunction

Beyond immediate autonomic effects, cold exposure can alter vascular function and increase cardiovascular workload (5, 36). Persistent vasoconstriction raises systemic vascular resistance and can increase cardiac afterload (30, 37). In a 2024 personal-level environmental temperature study, each 1 °C decrease was associated with increases of approximately 0.96 mmHg in 24-h systolic blood pressure and 0.28 mmHg in diastolic blood pressure among individuals with hypertension; the corresponding increases were smaller among normotensive individuals (38). These blood-pressure findings are intermediate physiological outcomes and should not be interpreted as direct mortality effect estimates.

Sustained elevations in blood pressure may contribute to myocardial ischemia and cerebrovascular stress, particularly in susceptible individuals (37). Experimental evidence also indicates that cold exposure can increase oxidative stress and inflammatory signaling in endothelial cells (39). Such changes may impair vascular homeostasis and contribute to a prothrombotic environment, although their role as direct mediators of population-level cardiovascular mortality remains uncertain.

These vascular alterations are particularly important in individuals with pre-existing atherosclerosis, whose impaired endothelial function and limited vascular reserve increase susceptibility to cold-induced cardiovascular events (5).

### 3.3 Hemorheological alterations and prothrombotic responses

Experimental studies also indicate that substantial cooling can alter blood rheology. For example, an *in vitro* study comparing blood samples at 36.5 °C and 22 °C reported increased whole-blood and plasma viscosity and reduced erythrocyte deformability at the lower temperature (40). These conditions represent a marked experimental manipulation and should not be interpreted as the blood temperatures ordinarily reached during community exposure to cold weather. The findings demonstrate temperature sensitivity of hemorheological properties rather than the magnitude of change expected during real-world ambient cold exposure.

Human experiments using controlled surface cooling have reported acute increases in hematocrit, platelet count, blood viscosity, and arterial pressure (41). These laboratory responses support the biological plausibility of hemoconcentration and altered thrombotic potential during cold stress, but their magnitude and persistence during ordinary winter exposure remain uncertain. The experiments also do not demonstrate that these hematologic changes mediate the population-level association between ambient cold exposure and cardiovascular mortality.

Hypertension, endothelial dysfunction, altered blood rheology, and prothrombotic responses may represent interacting components of the cardiovascular response to cold rather than completely independent processes. Experimental and physiological studies suggest that these responses could increase cardiovascular stress and may be particularly relevant in individuals with pre-existing cardiovascular disease or limited physiological reserve. However, the extent to which these experimentally observed changes occur during typical ambient winter exposure, and whether they causally mediate myocardial infarction, stroke, heart failure, sudden cardiac death, or other fatal cardiovascular outcomes, has not been established directly.

More broadly, most mechanistic studies have examined acute, controlled cold exposures that differ substantially from real-world community exposure in intensity, duration, body surface area exposed, clothing, physical activity, indoor–outdoor transitions, and opportunities for behavioral adaptation. Mechanistic findings should therefore be interpreted as evidence of physiological plausibility rather than as direct estimates of population exposure effects or demonstrated mediators of cold-related cardiovascular mortality.

### 3.4 Mechanistic integration and implications

Taken together, experimental and physiological evidence supports several biologically plausible pathways through which cold exposure may increase cardiovascular stress (5, 6, 28, 31, 37, 39–41). Sympathetic activation, vasoconstriction, blood-pressure elevation, endothelial dysfunction, hemorheological alterations, and prothrombotic responses may interact during cold exposure. These studies support mechanistic plausibility but do not independently establish an effect on population-level cardiovascular mortality, which is evaluated separately using epidemiological studies with mortality endpoints.

These physiological responses are unlikely to be uniform across populations. Repeated cold exposure can produce physiological acclimatization, whereas aging and baseline cardiovascular health may modify tolerance to acute cold stress (42–44). Behavioral and social or infrastructural adaptations may instead modify the degree of personal cold exposure and the capacity to prevent or respond to adverse events. Consequently, similar changes in ambient temperature may have different physiological and clinical implications across climatic and socioeconomic settings.

This integrated mechanistic perspective provides the biological foundation for understanding the regional heterogeneity in cold-related cardiovascular mortality discussed in the following sections. Figure 1 integrates these mechanistic findings while distinguishing experimentally and physiologically supported acute responses from proposed or indirect links to clinical cardiovascular events and population-level mortality. Future studies integrating experimental physiology with large-scale epidemiological investigations will be essential to clarify how environmental exposure, biological adaptation, and societal factors may jointly shape cardiovascular vulnerability during cold weather (43).

**Figure 1:**
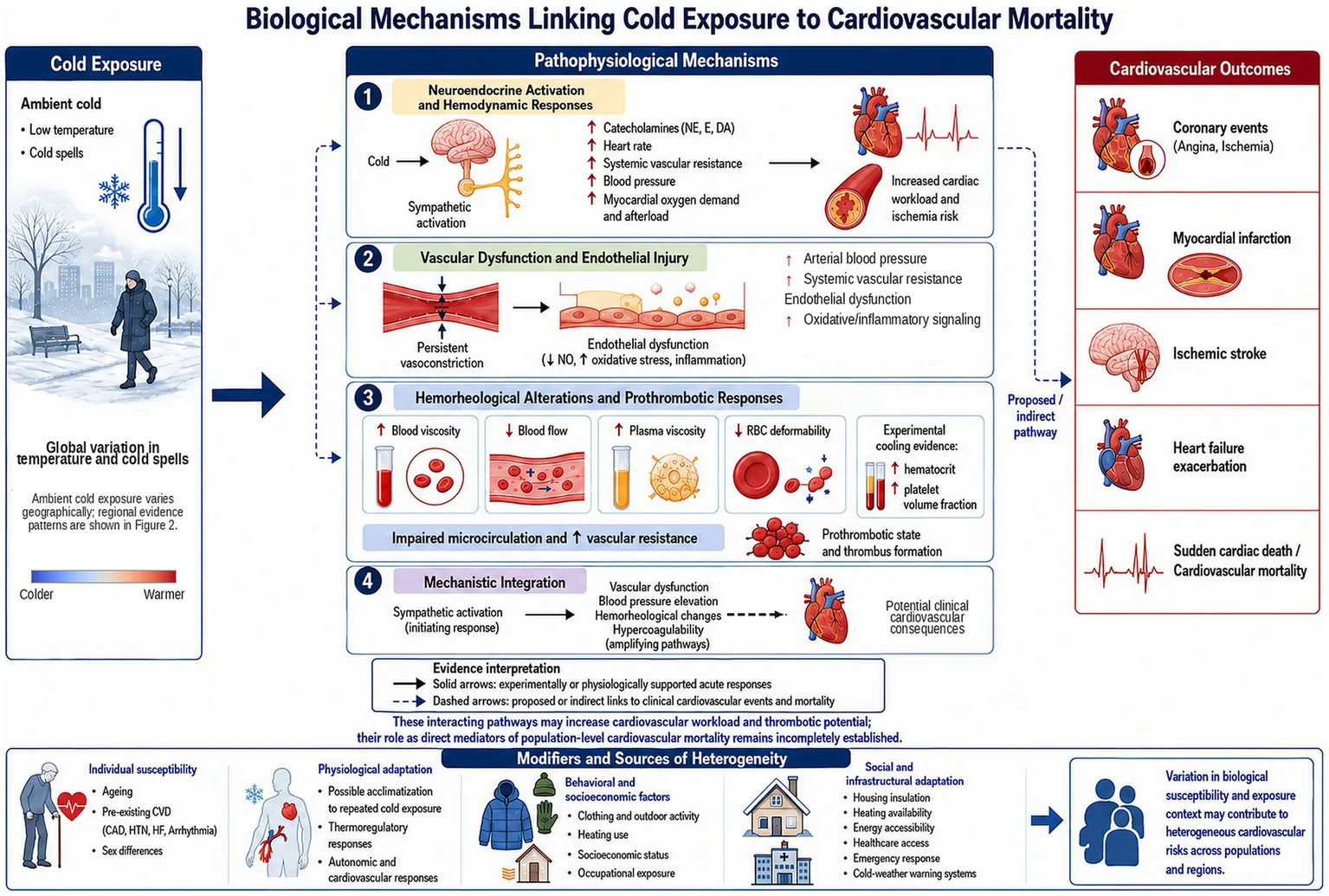
Biological mechanisms potentially linking cold exposure to adverse cardiovascular outcomes. Cold exposure may activate sympathetic and neuroendocrine responses, promote vasoconstriction and endothelial dysfunction, and induce hemorheological and prothrombotic alterations. Experimental and physiological studies support the biological plausibility of these pathways, but many are based on acute controlled cold exposures that differ from ordinary community exposure to winter temperatures. These mechanisms should therefore not be interpreted as demonstrated mediators of population-level cold-related cardiovascular mortality. The magnitude of physiological responses may vary according to individual susceptibility, possible physiological acclimatization, behavioral and socioeconomic factors, and social and infrastructural adaptation. The figure is a conceptual synthesis and does not represent a single experimentally established causal pathway or dataset. In the figure, solid arrows denote experimentally or physiologically supported acute responses, whereas dashed arrows denote proposed or indirect links to clinical cardiovascular events or population-level mortality that have not been established as direct causal mediators. CVD, cardiovascular disease; CAD, coronary artery disease; HTN, hypertension; HF, heart failure; NO, nitric oxide; RBC, red blood cell.

## 4 Global regional heterogeneity in cold-related cardiovascular mortality

For this review, climatic setting and socioeconomic context were treated as conceptually distinct dimensions rather than combined into a single regional classification. Climatic setting was characterized primarily by local temperature distributions, seasonal patterns, and study-specific definitions of cold rather than by latitude alone. Where relevant, altitude and other local geographic characteristics were also considered. Socioeconomic context was evaluated separately. National income level was classified according to the World Bank FY2026 income classification, based on 2024 gross national income per capita calculated using the Atlas method (45). Economies were classified as low income (≤US$1,135), lower-middle income (US$1,136–4,495), upper-middle income (US$4,496–13,935), or high income (>US$13,935). The collective term low- and middle-income countries (LMICs) refers to the low-, lower-middle-, and upper-middle-income categories. Income classification was used only as a standardized contextual descriptor and not as a proxy for housing quality, heating availability, healthcare accessibility, or population-level adaptive capacity.

In addition, epidemiological estimates reported across regions were derived from heterogeneous exposure definitions and effect measures. Accordingly, risk estimates per unit temperature decrease, cold-attributable fractions, excess deaths, and mortality changes during cold spells are presented as complementary indicators of cold-related cardiovascular burden rather than as directly comparable measures of effect magnitude.

Specifically, risk estimates per unit decrease in temperature describe the slope of an exposure–response relationship, cold-spell estimates quantify changes in risk during defined episodic events, attributable fractions estimate the proportion of deaths associated with cold exposure within a given population and exposure distribution, and excess-death estimates represent an absolute population burden. These measures therefore address different epidemiological questions and should not be used to rank regional vulnerability on a common numerical scale. Overall, 26 epidemiological studies contributed to the regional mortality synthesis. The evidentiary basis was uneven across settings and varied substantially in study design, geographic coverage, exposure assessment, lag specification, covariate adjustment, and statistical modeling. Because no formal study-level risk-of-bias score was applied, evidentiary strength was interpreted qualitatively. Greater weight was given to large multi-location, nationwide, and within-country studies using standardized or internally consistent analytical frameworks, whereas single-location and ecologically comparative findings were treated as more context-specific evidence. Characteristics and methodological differences of all 26 included epidemiological studies are summarized in Table 1.

### 4.1 Colder-climate settings

Evidence from colder-climate settings includes large multi-country, multi-location, nationwide, and population-based studies as well as more geographically restricted investigations. These studies support an association between cold exposure and cardiovascular or cause-specific cardiovascular mortality, although the magnitude of reported effects varies with temperature metric, exposure definition, lag structure, outcome definition, covariate adjustment, and analytical approach (11, 12, 46). Prolonged winters and low absolute temperatures therefore do not imply a uniform level of cold-related cardiovascular mortality. Observed differences may reflect exposure characteristics, population susceptibility, adaptive capacity, contextual factors, and methodological variation.

In the United States, a county-level analysis demonstrated a nonlinear temperature–CVD mortality relationship and found that cold accounted for most of the temperature-attributable cardiovascular burden (12). Cross-regional European and North American studies have likewise reported higher cardiovascular or cause-specific mortality at lower temperatures, although exposure metrics and analytical methods differ among studies (17, 18, 21, 46).

A substantial proportion of cold-related cardiovascular mortality may arise from the non-extreme portion of the cold temperature range rather than from rare episodes of extreme cold alone. In a seven-city Norwegian time-series analysis, 11.4% (95% CI 6.0–15.4%) of cardiovascular deaths were attributable to temperatures below the city-specific optimum temperature over lag 0–14 days, whereas approximately 0.3% were attributable to extreme cold (<1st percentile) (47). This finding concerns attributable burden within the study-specific temperature distribution and should not be interpreted as a universal threshold for “moderate cold.”

The comparatively moderate cold-related burden observed in some colder-climate settings may partly reflect behavioral, social, infrastructural, and possibly physiological adaptation. However, population-level epidemiological studies generally cannot distinguish the independent contribution of physiological acclimatization from correlated factors such as housing, heating, socioeconomic resources, healthcare capacity, and population structure (21, 48, 49). Cold-related cardiovascular mortality nevertheless remains evident in well-resourced populations, and older adults or people with pre-existing cardiovascular disease may be more susceptible (10, 50, 51).

### 4.2 Mild-winter settings

Cold-related cardiovascular mortality is also reported in settings with relatively mild winters. Evidence from China, Brazil, Thailand, and India demonstrates that adverse cardiovascular or cause-specific mortality associations can occur at temperatures that are not extreme in absolute terms (13, 52–54). These studies use different exposure definitions and effect measures, however, and they do not establish that mild-winter populations are inherently more vulnerable than colder-climate populations.

Several multi-location studies from China demonstrate substantial within-country heterogeneity. In 15 Chinese megacities, 15.8% (95% empirical CI 13.1–17.9%) of cardiovascular deaths were attributable to temperatures below city-specific minimum mortality temperatures over lag 0–21 days (13). A separate analysis of 26 regions also reported marked variation in low-temperature-associated CVD mortality (20). North–south differences should be interpreted cautiously because air pollution, socioeconomic conditions, housing and heating, healthcare accessibility, population structure, and other seasonal co-exposures may vary concurrently. Differences in diagnostic capacity, death certification, and cause-of-death classification may also contribute to apparent regional variation; climatic adaptation or absolute temperature alone therefore cannot be assumed to explain the observed pattern (55, 56).

Using a different burden metric, an ecological time-series analysis in Brazil estimated approximately 661 cold-attributable acute myocardial infarction deaths per year in São Paulo, with cumulative lags of up to 14 days (52). This is an absolute excess-death estimate and should not be compared numerically with attributable fractions or relative risks from other settings. Taken qualitatively, studies from mild- and warm-climate settings show that cold-related cardiovascular mortality can occur outside traditionally cold regions, but the available evidence is insufficient to establish a systematic difference in susceptibility by climate or socioeconomic resources.

Collectively, these findings indicate that cold-related cardiovascular mortality can occur in mild-winter settings, but they do not establish that populations in such settings are inherently more vulnerable. Housing insulation, heating availability, socioeconomic circumstances, healthcare accessibility, and other contextual factors may modify individual cold exposure or cardiovascular risk; however, their contributions to the observed regional differences have not been established consistently (50, 52).

### 4.3 Warm-climate settings: limited evidence

Evidence from warm-climate settings remains comparatively sparse, particularly in Africa, South Asia, Southeast Asia, and parts of Latin America (57–59). Nevertheless, available studies demonstrate that cold-related mortality can occur in warm or lower-latitude populations. Nationally representative evidence from India identified cold-attributable mortality from ischemic heart disease and stroke (54), while studies in Thailand have reported temperature-related CVD mortality at national or multi-province scales (53, 60). These findings establish the presence of cold-related health effects in selected warm-climate settings but do not demonstrate that warm-climate populations as a whole are more vulnerable.

Interpretation should therefore remain cautious. Large areas of Africa, South Asia, and Latin America are still underrepresented in the cold–cardiovascular literature (58, 59). The scarcity of studies should not be interpreted as evidence of either greater or lower population vulnerability. For example, a nationwide study across six regions of Thailand used nine station-specific percentile-based cold-wave definitions requiring low temperatures for 2–4 consecutive days and reported increased CVD mortality during cold-wave periods (RR 1.076, 95% CI 1.061–1.090; principal pooled estimate at lag 0–3 days) (61). This event-based relative risk is conceptually distinct from cold-attributable fractions or per-degree exposure–response estimates. More geographically diverse studies using standardized methods are needed before regional vulnerability can be characterized reliably.

Figure 2 provides a conceptual synthesis of the regional evidence discussed above. Rather than ranking populations by vulnerability, it illustrates the uneven geographic distribution of the evidence, selected context-specific epidemiological findings, and the climatic, socioeconomic, adaptive, seasonal, and methodological factors that may contribute to observed heterogeneity. Table 1 presents the characteristics and reported estimates of all 26 epidemiological studies included in the regional mortality synthesis.

**Figure 2:**
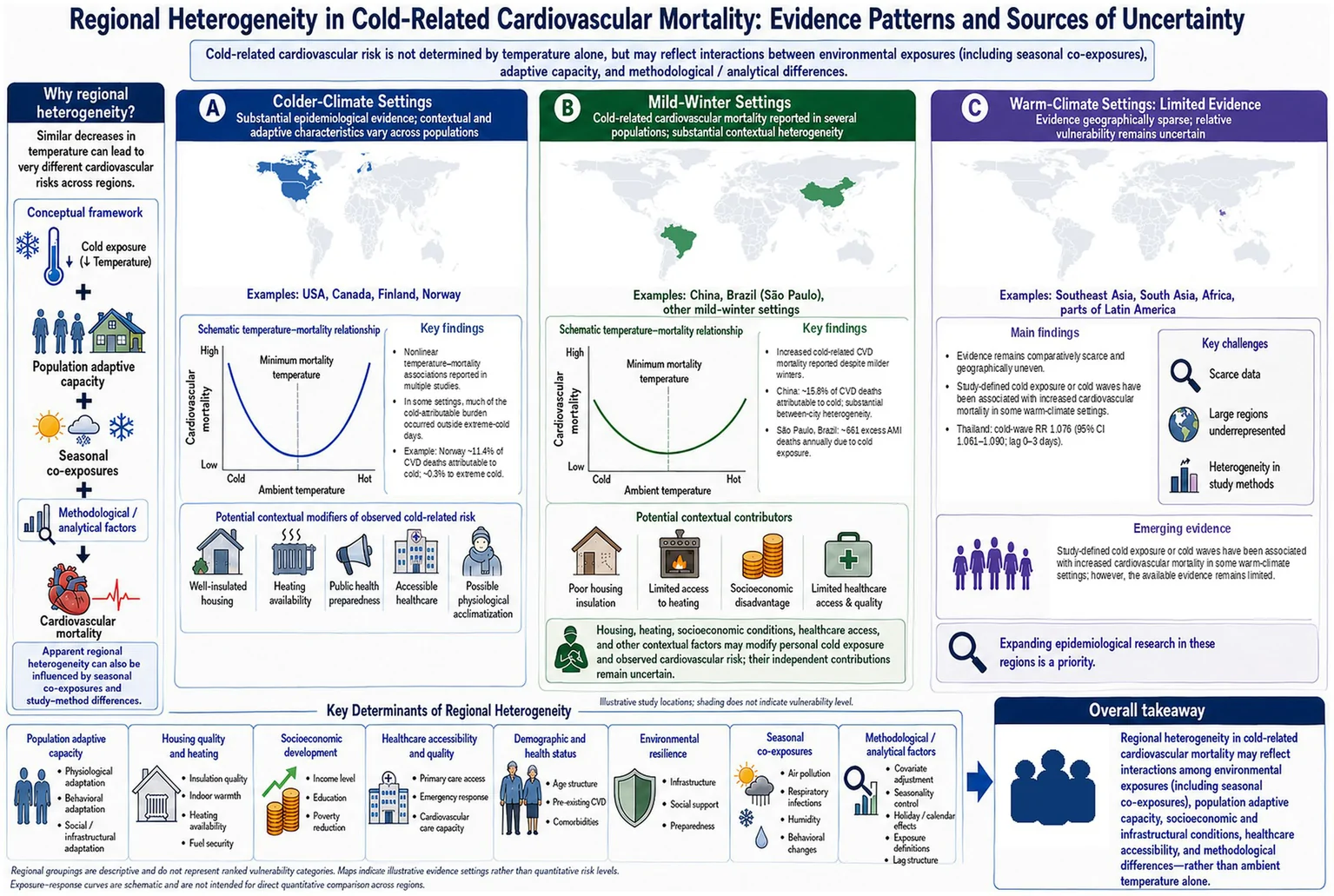
Regional heterogeneity in cold-related cardiovascular mortality: evidence patterns and sources of uncertainty. The conceptual framework distinguishes three principal population-level dimensions that may contribute to regional heterogeneity: environmental exposure, population adaptation and susceptibility, and societal resilience, including socioeconomic conditions, housing and heating infrastructure, and healthcare accessibility. Seasonal co-exposures, including air pollution, respiratory infections, humidity, and behavioral changes, may additionally influence observed cold-related cardiovascular risk. Methodological and analytical differences among studies, including covariate adjustment, control of seasonality, holiday or calendar effects, exposure definitions, and lag specification, may further contribute to apparent regional heterogeneity. These components are presented as potential contributors to observed regional variation rather than as established causal determinants. The regional groupings are descriptive and should not be interpreted as ranked categories of cardiovascular vulnerability. Map shading indicates illustrative geographic settings represented in the evidence base rather than quantitative levels of cold-related risk. Evidence remains limited in many warm-climate regions, and relative vulnerability in these settings remains uncertain.

### 4.4 Comparative synthesis

Taken together, the available evidence supports geographic heterogeneity in cold-related cardiovascular mortality but does not justify a simple ranking of population vulnerability across countries. The strongest evidence for cross-regional heterogeneity comes from multi-country or within-country analyses in which locations are evaluated using common or internally consistent analytical frameworks (11, 49, 62). Country-specific estimates derived from independent studies—including attributable fractions, relative risks, excess deaths, and cold-spell effects—should instead be interpreted as context-specific indicators of burden rather than directly compared as measures of relative vulnerability.

Within standardized or internally comparable analyses, variation in cold-related mortality has been observed across locations with different climatic and contextual characteristics. Such heterogeneity may be associated with differences in local temperature distributions, housing and heating conditions, socioeconomic circumstances, healthcare capacity, behavioral responses, and population-level adaptive capacity. However, these factors are correlated, and existing observational evidence generally does not permit their independent contributions to regional differences to be established. Cross-regional patterns should therefore be interpreted as evidence of heterogeneity rather than as proof that one climatic or socioeconomic setting is inherently more vulnerable than another.

Climate change may further modify regional patterns of cold-related cardiovascular mortality through changes in cold exposure, population susceptibility, and adaptive capacity; these implications are considered further in the Discussion. These potential future changes should be distinguished from the observed cold-related mortality associations summarized in the present review, and projections should not be interpreted as evidence that cold events will increase uniformly across regions.

## 5 Determinants of regional heterogeneity

Several competing and potentially overlapping explanations have been proposed for the observed regional variation in cold-related cardiovascular mortality. These include differences in physiological susceptibility and acclimatization, personal cold exposure resulting from housing and heating conditions, behavioral responses, socioeconomic circumstances, healthcare accessibility, seasonal co-exposures, and methodological differences among studies. A central challenge is that these factors are strongly correlated within populations, making their independent contributions difficult to distinguish in most observational studies. The following sections therefore consider each potential determinant according to the directness of the available evidence rather than assuming that any single factor explains the observed geographic patterns.

When interpreting potential determinants of regional heterogeneity, greater weight was given to studies that directly evaluated effect modification, stratified associations, within-country contrasts, or policy-related differences. Relevant evidence includes analyses of demographic and socioeconomic characteristics, urban–rural residence, housing and heating conditions, and related contextual factors (10, 19, 20, 63–66). Cross-country or cross-region correlations were treated as supportive or hypothesis-generating evidence rather than proof that a contextual characteristic caused the observed mortality difference.

### 5.1 Climatic adaptation and population-level adaptive capacity

Long-term climatic adaptation has been proposed as one potential contributor to regional heterogeneity in cold-related cardiovascular mortality. In this review, population-level adaptive capacity is conceptualized as comprising three distinct but interacting dimensions: physiological, behavioral, and social/infrastructural adaptation. Physiological adaptation refers to biological acclimatization resulting from repeated or prolonged cold exposure, including potential changes in thermoregulatory, autonomic, and cardiovascular responses. Behavioral adaptation includes individual responses such as changes in clothing, outdoor activity, and heating use. Social and infrastructural adaptation includes housing insulation, heating systems, energy accessibility, cold-weather warning systems, healthcare services, and other population-level protective resources.

These dimensions should not be regarded as interchangeable. In particular, widespread access to heating and better housing quality may reduce actual cold exposure but should not be interpreted as evidence of physiological adaptation. Moreover, the available population-level epidemiological evidence does not permit the independent contribution of physiological adaptation to be clearly distinguished from behavioral and social/infrastructural adaptation. Accordingly, climatic adaptation is considered here within the broader concept of population-level adaptive capacity rather than as an independently established causal determinant.

A seven-city Norwegian analysis indicates that persistent non-extreme cold can account for a substantial share of temperature-attributable cardiovascular mortality (47). Multi-country analyses also show that temperature-related vulnerability varies across cities and is associated with climatic and urban characteristics (49). In a separate case-crossover study from Oulu, Finland, each additional cold day during a cold-spell exposure window was associated with an increased risk of sudden cardiac death (67). These estimates are not quantitatively equivalent, but they support the epidemiological relevance of exposure duration and persistence.

Populations in regions with relatively mild or short winters may experience different patterns of cold exposure and behavioral protection than populations in colder settings, but physiological acclimatization has rarely been measured directly in mortality studies. Evidence from India and Thailand demonstrates adverse cardiovascular or cause-specific mortality associations with moderate cold or study-defined cold waves (54, 61). Because these studies use different exposure definitions and effect measures, their numerical estimates should not be compared directly. They provide qualitative evidence that cold-related cardiovascular mortality can occur in relatively mild or warm climates, not proof that reduced acclimatization is the cause.

Overall, available findings are consistent with the possibility that population-level adaptive capacity contributes to geographic variation in cold-related cardiovascular mortality, but adaptation should not be treated as a single causal explanation. Physiological acclimatization, behavioral responses, housing and heating, socioeconomic conditions, healthcare resources, and other correlated factors are difficult to separate in existing observational studies (21, 44, 49).

### 5.2 Socioeconomic development, housing, and heating

Socioeconomic context was considered independently from climatic setting because populations exposed to similar outdoor temperatures can differ substantially in income, housing quality, heating access, energy security, and healthcare resources (48, 49). Housing insulation and heating can modify personal cold exposure by altering the indoor thermal environment. Among older adults in Japan, indoor temperature was more strongly associated with ambulatory blood pressure during colder months than outdoor temperature (68), supporting the relevance of indoor thermal conditions to cardiovascular physiology.

Evidence from indoor-temperature studies and intermediate cardiovascular outcomes suggests that colder indoor environments may adversely affect cardiovascular physiology (69–71). These studies support the plausibility that housing and indoor thermal conditions modify cardiovascular responses to cold exposure, but they do not by themselves establish an effect on cardiovascular mortality.

Separate population-based and policy studies have examined mortality outcomes in relation to housing and heating conditions. These studies were given greater interpretive weight than cross-country ecological comparisons because they directly compared mortality or cardiovascular outcomes across populations with different heating, housing, or related exposure conditions.

Housing and heating have also been evaluated in studies with mortality or major cardiovascular outcomes. Improved insulation and heating have been associated with lower mortality risk in vulnerable populations, while heating-fuel studies have reported differences in mortality and cardiovascular events (65, 72). In China, a national analysis of 364 locations found that central heating was associated with lower winter mortality and that each one-week reduction in heating duration was associated with a 3.32% increase in total mortality risk (66). Because the endpoint was overall winter mortality rather than cold-attributable cardiovascular mortality specifically, this evidence supports heating as a contextual modifier rather than proving an independent cardiovascular mortality effect.

Importantly, these findings primarily concern overall or winter mortality and should therefore be interpreted as evidence that heating infrastructure may represent an important contextual modifier, rather than as direct evidence that heating independently determines cold-related cardiovascular mortality.

In some mild-winter or resource-constrained settings, limited heating infrastructure, inadequate housing insulation, and energy insecurity may increase personal cold exposure despite comparatively moderate outdoor temperatures. However, direct epidemiological evidence linking these housing and heating characteristics specifically to cardiovascular mortality remains limited in many regions, particularly in Africa, South Asia, and parts of Latin America. Housing and heating conditions are therefore interpreted in this review as plausible social and infrastructural modifiers rather than as established causal explanations for regional differences in cold-related cardiovascular mortality.

### 5.3 Healthcare resources

Healthcare resources may plausibly modify cold-related cardiovascular vulnerability by influencing the recognition, treatment, and survival of acute cardiovascular events. Timely diagnosis, emergency transportation, specialist cardiovascular treatment, and post-event clinical management may therefore be relevant contextual factors when interpreting differences among populations.

Most population-level temperature–mortality studies do not directly measure healthcare accessibility, emergency response capacity, treatment delay, or specialist availability as mediators of the cold–cardiovascular mortality association. Multi-country analyses can identify geographic heterogeneity across healthcare environments, but the contribution of healthcare resources cannot usually be separated from socioeconomic, infrastructural, and population characteristics (4, 49). Healthcare capacity is therefore treated here as a plausible contextual modifier rather than an established causal determinant.

Within-country evidence may nevertheless provide indirect support for the relevance of healthcare context. For example, higher cold-related myocardial infarction mortality has been reported in rural than in urban populations in China (64), where differences in healthcare access may coexist with differences in socioeconomic conditions, housing, occupational exposure, population structure, and other contextual characteristics. These correlated factors cannot be disentangled reliably in the available population-level evidence. Healthcare resources are therefore interpreted in this review as plausible contextual modifiers rather than as established causal explanations for regional differences in cold-related cardiovascular mortality.

From a public health perspective, strengthening emergency cardiovascular care, primary healthcare capacity, cold-weather warning systems, and equitable access to medical services may still represent reasonable components of cold-related health protection. However, the effectiveness of these interventions in specifically reducing cold-attributable cardiovascular mortality requires more direct evaluation.

### 5.4 Individual susceptibility and environmental co-exposures

Individual susceptibility may modify the association between cold exposure and cardiovascular mortality. Age, sex, pre-existing cardiovascular disease, hypertension, and other cardiometabolic conditions can influence thermoregulatory capacity and cardiovascular responses to cold (10, 50, 51, 68). Older adults and people with established cardiovascular disease are frequently identified as higher-risk groups, although effect modification is not uniform across studies. Sex-specific differences have also been reported, with direction and magnitude varying by population and exposure definition (10, 61). Within-country analyses in China have identified heterogeneity by urban–rural residence and socioeconomic or demographic characteristics (20, 63, 64). Urban–rural residence is a composite contextual indicator, however, and does not identify which component—housing, heating, occupation, healthcare access, socioeconomic conditions, or population structure—causes the observed difference.

Physical activity and tobacco use may additionally influence cardiovascular susceptibility and the degree of personal cold exposure. In particular, exercise performed in cold environments can increase cardiovascular workload in susceptible individuals, whereas smoking may contribute to baseline vascular and cardiovascular risk (6). However, these behavioral factors have not been consistently measured or formally evaluated as effect modifiers in population-based studies, and their contribution to regional heterogeneity therefore remains uncertain.

Environmental co-exposures are another important consideration. Ambient air pollution, particularly particulate matter, is independently associated with cardiovascular morbidity and mortality and may co-occur with cold conditions (4, 73). Colder periods can also coincide with higher emissions from residential heating and reduced building ventilation, potentially increasing exposure to combustion-related pollutants (74, 75). Temperature may modify the health effects of air pollutants, although the direction and magnitude of interaction vary by pollutant, population, and climate (73, 76). A 2026 study from Shijiazhuang further reported elevated circulatory mortality during joint exposure to cold waves and PM2.5, supporting the need to consider compound environmental exposures (77). Respiratory infections may also contribute to seasonal cardiovascular risk. Influenza epidemics can trigger cardiac events and account for part of winter mortality, although adjustment for influenza does not necessarily remove the association between low temperature and cardiac mortality (78). Future studies should therefore model cold exposure within a broader seasonal and multi-exposure framework.

### 5.5 Interactions among determinants

The available evidence indicates that the determinants discussed above should not be regarded as independent risk factors but as components of an interconnected system linking environmental exposure to cardiovascular mortality. Within this system, adaptation may operate through three conceptually distinct but interacting pathways. Physiological adaptation may modify individual biological responses to repeated cold exposure. Behavioral adaptation may alter the intensity and duration of personal cold exposure through changes in clothing, outdoor activity, and heating use. Social and infrastructural adaptation encompasses housing insulation, heating infrastructure, energy accessibility, cold-weather warning systems, and healthcare capacity, all of which may modify personal exposure or the consequences of cold-related cardiovascular events (20). These pathways may interact but should not be interpreted as equivalent.

Contextual conditions in warm-climate or lower-resource settings may differ from those in colder or higher-resource settings, but these characteristics have not been measured consistently in cold-related cardiovascular mortality studies. Cross-country analyses of winter mortality and urban vulnerability suggest that housing, heating, socioeconomic conditions, and other urban characteristics may matter (48, 49), but such findings remain partly ecological. These factors should therefore be treated as plausible modifiers requiring direct investigation rather than as evidence that warm-climate or lower-latitude populations are inherently more vulnerable.

Regional heterogeneity in cold-related cardiovascular mortality may reflect interactions among environmental exposure, population adaptation, and societal resilience. Seasonal co-exposures and methodological differences may also alter estimated associations. For example, regional analyses in China, influenza-adjusted cardiac mortality studies, and studies comparing alternative cold-spell definitions demonstrate that exposure definitions, covariate adjustment, and model choices can materially affect reported estimates (20, 78, 79). These methodological influences should be distinguished from underlying population susceptibility.

This integrative framework provides a basis for interpreting geographic heterogeneity without assuming that a universal temperature threshold applies across populations. Multi-region European evidence and cold-warning research both support the use of locally calibrated temperature–health relationships rather than a single absolute threshold (21, 80). Future research should investigate climatic conditions, indoor thermal environments, housing quality, energy poverty, healthcare accessibility, and chronic disease burden using harmonized multi-location designs.

## 6 Discussion

### 6.1 Principal findings and unresolved questions

The available evidence supports genuine geographic heterogeneity in cold-related cardiovascular mortality, but the sources of this heterogeneity remain only partly resolved. A key unresolved question is the extent to which observed between-region differences represent true effect modification by population susceptibility and adaptation, as opposed to differences in exposure measurement, study design, analytical specification, seasonal co-exposures, or outcome ascertainment.

Several competing explanations remain plausible. First, physiological acclimatization and differences in baseline cardiovascular susceptibility may modify biological responses to cold. Second, housing quality, insulation, heating use, clothing, and behavioral responses may alter actual personal cold exposure even when populations experience similar outdoor temperatures. Third, socioeconomic conditions and healthcare accessibility may influence both exposure and survival following acute cardiovascular events. Fourth, seasonal co-exposures, including air pollution, respiratory infections, humidity, changes in physical activity, and other winter-related behaviors, may confound or modify observed temperature–mortality associations. Finally, differences in temperature metrics, cold-spell definitions, lag structures, model specification, death certification, and diagnostic capacity may create apparent geographic heterogeneity even when underlying biological susceptibility is similar.

The current evidence does not allow these explanations to be separated reliably. Most epidemiological studies measure outdoor ambient temperature rather than individual thermal exposure, while housing, heating, behavioral adaptation, healthcare access, and co-exposures are rarely measured simultaneously.

This reliance on outdoor temperature may introduce substantial exposure misclassification because outdoor conditions do not necessarily correspond to the temperatures actually experienced by individuals. The relationship between outdoor and personal thermal exposure may differ according to housing insulation, heating use, energy affordability, time spent indoors, occupational activity, and behavioral responses to cold. Consequently, the same outdoor temperature may represent substantially different levels of personal cold exposure across populations, potentially contributing to apparent regional heterogeneity in temperature–mortality associations. Future multi-location studies should therefore combine outdoor meteorological data with repeated indoor temperature measurements, personal temperature monitoring where feasible, time–activity information, and standardized indicators of housing quality, heating use, and energy insecurity. Such data would allow investigators to distinguish the effects of ambient climatic conditions from differences in actual personal cold exposure and to test whether indoor thermal conditions mediate or modify cold-related cardiovascular mortality.

Moreover, many candidate determinants are correlated at both individual and population levels. Consequently, associations between a regional characteristic and cold-related mortality should not be interpreted as evidence that the characteristic independently explains the observed difference. Resolving these competing explanations will require harmonized multi-location studies that combine standardized temperature–mortality modeling with individual- or area-level information on indoor thermal conditions, housing and heating, socioeconomic circumstances, healthcare access, air pollution, respiratory infections, and population susceptibility.

Future multi-location studies could operationalize the proposed framework using hierarchical or multilevel models in which environmental exposure, population adaptation, and societal resilience are represented by prespecified indicators measured at individual, community, and regional levels. Variance-partitioning, sequential model comparison, and cross-level interaction analyses could then be used to estimate the incremental contribution of each component and to determine whether adaptation or resilience modifies the temperature–mortality association after accounting for climatic exposure and major confounders. Where longitudinal or quasi-experimental data are available, mediation or decomposition approaches and natural experiments involving changes in heating provision, housing conditions, or healthcare access could further help distinguish pathways related to exposure reduction from those related to physiological susceptibility or post-event survival.

### 6.2 Biological and societal determinants

Biological susceptibility and social or infrastructural conditions may operate at different stages of the pathway from cold exposure to fatal cardiovascular outcomes. Experimental evidence supports cold-induced sympathetic activation, blood-pressure elevation, vasoconstriction, endothelial inflammatory signaling, hemorheological changes, and prothrombotic responses (5, 28, 37, 39–41). The extent to which these responses contribute to population-level mortality is likely to depend on actual personal cold exposure, baseline cardiovascular susceptibility, and contextual conditions affecting prevention, treatment, and survival.

Observational evidence suggests that housing, heating, socioeconomic circumstances, and healthcare context may contribute to differences in cold-related cardiovascular risk, but current studies do not support broad regional generalizations regarding these characteristics. Within-country analyses may provide more informative contrasts because populations can be compared within a more consistent national context. For example, urban–rural differences in cold-related myocardial infarction mortality have been reported in China (64); however, healthcare accessibility in such comparisons is strongly correlated with housing, occupation, socioeconomic conditions, population structure, and other contextual factors. The observed difference therefore cannot be attributed specifically to healthcare access.

In addition, some apparent between-region differences may partly reflect variation in death certification practices, diagnostic capacity, and the recording or classification of cardiovascular causes of death across healthcare systems.

An important unresolved issue is whether biological susceptibility and social or infrastructural conditions act independently, sequentially, or interactively. For example, inadequate heating may increase personal cold exposure, whereas limited healthcare access may influence survival after a cardiovascular event; these mechanisms operate at different stages of the pathway from environmental exposure to mortality. Existing population-level studies rarely measure these processes simultaneously, making it difficult to determine which stage contributes most to observed regional differences. Future studies should therefore distinguish determinants of exposure from determinants of physiological response and determinants of post-event survival.

This distinction could be tested by linking repeated sensor-based measurements of indoor and, where feasible, personal thermal exposure and physiological responses with population-level mortality data and indicators of healthcare access, thereby allowing the exposure, susceptibility, and post-event survival components of the framework to be evaluated separately.

### 6.3 Public health implications

The findings of this review have implications for cold-health policy and climate adaptation. Fixed meteorological thresholds may not perform equally well across populations because temperature–health relationships vary by climate, local exposure distribution, and population characteristics. Cold-specific evidence shows that warning thresholds, indicators, and time windows affect the identification of cardiovascular risk periods, while operational experience from Quebec demonstrates the feasibility of linking meteorological surveillance with locally defined cold-health criteria (80, 81). Broader public-health guidance has similarly emphasized the need to adapt cold-protection strategies to local vulnerability (82).

The practical implementation of cold-health early-warning systems should extend beyond issuing alerts for isolated extreme cold events. In colder-climate settings, where prolonged or persistent moderate cold may account for a substantial proportion of cardiovascular burden, surveillance systems should also consider the duration and cumulative intensity of cold exposure. Locally calibrated warning frameworks could integrate short-term meteorological forecasts with locally validated temperature thresholds, exposure duration, lag structures, and indicators of population vulnerability, including age structure, cardiovascular disease prevalence, housing and heating conditions, energy insecurity, and healthcare accessibility. Warning levels could then be linked to predefined responses, such as targeted communication to older adults and individuals with cardiovascular disease, community outreach, support for adequate indoor heating, and enhanced preparedness of primary care and emergency cardiovascular services.

Although evidence derived from heat should not be extrapolated as evidence of cold-specific cardiovascular effects, recent temperature-health research provides useful methodological principles for locally adapted warning systems. A global systematic review and meta-analysis of locally defined extreme-heat events illustrates the importance of considering locally defined exposure thresholds and differences in population vulnerability and adaptation across settings (83). Similarly, recent work using data-driven short-term heatwave forecasting in an arid, data-sparse setting demonstrates how environmental surveillance and predictive modeling can be integrated to provide advance warning of hazardous temperature events (84). These studies are relevant to the general principles of local calibration and predictive environmental surveillance but are not interpreted here as evidence regarding cold-related cardiovascular mortality.

Taken together, these findings support the development of region-specific cold-risk assessment and early-warning frameworks that integrate climatic conditions with socioeconomic indicators, housing quality, heating availability, healthcare accessibility, and population vulnerability (20). Such systems should be locally developed and periodically validated against observed health outcomes because population adaptation, infrastructure, demographic composition, and temperature–health relationships may change over time. Linking locally calibrated environmental surveillance with clearly defined public health actions may improve identification of high-risk periods and populations and facilitate more effective allocation of preventive and healthcare resources.

### 6.4 Strengths, limitations, and future research priorities

A major strength of the present review is its integration of epidemiological evidence across diverse climatic regions to develop a unified conceptual framework explaining regional heterogeneity in cold-related cardiovascular mortality. Rather than considering ambient temperature as an isolated environmental exposure, this review synthesizes evidence from biological, environmental, and societal perspectives, thereby providing a broader understanding of the mechanisms through which cold exposure influences cardiovascular health.

Several limitations should be acknowledged. First, the geographic distribution of evidence is highly uneven. Studies remain concentrated in North America, Europe, and China, whereas Africa, South Asia, and parts of Latin America are underrepresented (58, 59). This evidence gap should not be interpreted as proof of either greater or lower cardiovascular vulnerability to cold. Second, substantial methodological heterogeneity exists in temperature metrics, cold-spell definitions, exposure duration, lag structures, and effect measures (56, 79, 80). Per-degree risks, cold-attributable fractions, excess deaths, and cold-spell relative risks are not equivalent and should not be compared as if they measured the same quantity. Cross-regional comparisons may also be influenced by diagnostic capacity, death certification, and cause-of-death classification. Third, most temperature–mortality studies use outdoor ambient temperature as a proxy for personal exposure. Indoor-temperature and personal-exposure studies demonstrate why this proxy can be imperfect, particularly when housing, heating use, energy affordability, and time spent indoors differ across populations (68, 69, 71). Finally, adjustment for individual cardiovascular risk factors and seasonal co-exposures is inconsistent. Cold periods may coincide with influenza, air pollution, humidity changes, altered physical activity, medication-related factors, and holiday or calendar effects. Influenza adjustment, for example, does not necessarily eliminate the low-temperature association with cardiac mortality (78).

Studies also differ in how they control seasonality, air pollution, influenza activity, humidity, day of the week, holidays, long-term trends, and lag structure. Multi-location and cold-spell studies show that analytic specification can influence estimated temperature effects (49, 78, 79). Consequently, some between-study differences may reflect co-exposure patterns or model specification rather than true differences in population susceptibility. Residual confounding and unmeasured effect modification therefore remain important limitations.

Fourth, literature selection and data extraction were conducted by one reviewer without duplicate independent screening or extraction, and methodological quality was assessed qualitatively rather than with design-specific risk-of-bias tools. These features may increase susceptibility to selection and interpretive bias.

Finally, most available studies emphasize short-term mortality effects, whereas the long-term cumulative consequences of repeated cold exposure and dynamic changes in adaptation remain less well characterized (59).

Future research should move beyond conventional ambient-temperature models by integrating indoor thermal conditions, housing quality, energy poverty, healthcare accessibility, behavioral adaptation, chronic disease burden, and concurrent environmental exposures. Where data permit, models should jointly evaluate temperature with major seasonal co-exposures, including air pollution, influenza or other respiratory infection activity, and humidity, while considering behavioral changes, medication-related factors, heating-related emissions, and calendar effects (4, 73–78). Harmonization of covariate adjustment and lag specification across multi-location studies would improve interpretation of geographic heterogeneity.

Expanding research in underrepresented regions and adopting standardized exposure definitions are essential for improving cross-regional comparability and developing climate-adaptive strategies for reducing cold-related cardiovascular mortality (11, 58, 59).

Climate change is expected to alter the geographic distribution of cold-related cardiovascular risk rather than affect all regions uniformly. Climate projections indicate that the frequency and intensity of cold extremes will generally decrease with continued warming across most inhabited regions, although changes will vary geographically and cold spells will not disappear (85). Projection studies also suggest that future cold-related mortality will depend on both climatic change and adaptation (86). Consequently, some populations may experience less severe cold exposure, while the health burden of remaining cold events will also depend on demographic aging, baseline cardiovascular susceptibility, housing and heating, energy insecurity, healthcare capacity, and behavioral, social, and physiological adaptation. Region-specific projections should therefore combine climate-model outputs with locally calibrated temperature–mortality relationships and explicit assumptions about demographic and adaptive change.

## 7 Conclusion

Ambient cold exposure is consistently associated with increased cardiovascular mortality, but the magnitude and distribution of this burden vary substantially across populations. The available evidence supports geographic heterogeneity but does not justify a simple ranking of population vulnerability across climatic regions or socioeconomic groups, particularly because exposure definitions, effect measures, study designs, and geographic coverage remain heterogeneous.

The principal unresolved question is how much of the observed heterogeneity reflects true differences in physiological susceptibility and adaptation versus differences in personal thermal exposure, housing and heating conditions, healthcare access, seasonal co-exposures, and methodological or outcome-ascertainment factors. On the basis of the evidence gaps identified in this review, several priorities emerge. First, surveillance and epidemiological research should be expanded in currently underrepresented warm-climate and lower-resource regions, particularly in Africa, South Asia, Southeast Asia, and Latin America, without presuming that these populations are inherently more vulnerable to cold. Second, future multi-location studies should harmonize cold-exposure metrics, cold-spell definitions, lag structures, cardiovascular outcomes, and reporting of effect measures to improve cross-regional comparability. Third, exposure assessment should move beyond outdoor ambient temperature by incorporating repeated indoor and, where feasible, personal thermal measurements together with information on housing quality, heating use, energy insecurity, and time–activity patterns. Finally, public health practice should translate locally derived evidence into region-specific cold-health warning and prevention strategies that account for local temperature–mortality relationships, population susceptibility, housing and heating conditions, and healthcare capacity. Addressing these priorities would strengthen the interpretation of regional heterogeneity and support more targeted strategies to reduce cold-related cardiovascular mortality.
