# Supplementary material for: Environmental cold exposure and cardiovascular mortality: biological mechanisms, regional heterogeneity, and public health implications
Source: Front Public Health. 2026 Sep 30;14:1961371. doi: 10.3389/fpubh.2026.1961371 (PMC13662766; doi:10.3389/fpubh.2026.1961371)
Supplement: Supplementary file 1 [file Supplementary_file_1.DOCX]

Supplementary Material

## Supplementary Appendix S1. Database-specific search strategies

The search strategies were rerun on 2 September 2026 to document the final database syntax and confirm that the reported queries remained executable. Eligibility was restricted to publications published on or before 30 June 2026. Searches combined database-specific controlled vocabulary, where available, with title, abstract, or keyword terms. English- and Chinese-language publications were eligible. Reference-list screening supplemented the electronic searches. Original database-level retrieval and deduplication counts were not preserved in the contemporaneous search log. Counts from the later rerun were not substituted because they would reflect a later database-indexing state rather than the original screening denominator.

Table 1. Electronic databases and search information

| **Database** | **Platform** | **Search date** | **Coverage** | **Results** |
| --- | --- | --- | --- | --- |
| PubMed | NCBI | 2 Sep 2026 | Inception-30 Jun 2026 | Original count not preserved |
| Embase | Embase.com | 2 Sep 2026 | Inception-30 Jun 2026 | Original count not preserved |
| Web of Science | Core Collection | 2 Sep 2026 | Inception-30 Jun 2026 | Original count not preserved |
| Cochrane Library | Wiley | 2 Sep 2026 | Inception-30 Jun 2026 | Original count not preserved |
| CNKI | CNKI | 2 Sep 2026 | Inception-30 Jun 2026 | Original count not preserved |

Table 2. Detailed search strategies for each database

| **PubMed (NCBI)** | (("Cold Temperature"[Mesh] OR "Climate"[Mesh] OR "cold exposure"[Title/Abstract] OR "low temperature*"[Title/Abstract] OR "ambient temperature*"[Title/Abstract] OR "cold weather"[Title/Abstract] OR "cold spell*"[Title/Abstract] OR "cold wave*"[Title/Abstract]) AND ("Cardiovascular Diseases"[Mesh] OR "Cardiovascular System"[Mesh] OR cardiovascular[Title/Abstract] OR "cardiovascular disease*"[Title/Abstract] OR "heart disease*"[Title/Abstract] OR coronary[Title/Abstract] OR myocardial[Title/Abstract] OR stroke[Title/Abstract] OR cerebrovascular[Title/Abstract]) AND ("Mortality"[Mesh] OR mortalit*[Title/Abstract] OR death*[Title/Abstract] OR fatal*[Title/Abstract])) AND ("1900/01/01"[Date - Publication]: "2026/06/30"[Date - Publication]) |
| --- | --- |
| **Embase (Embase.com)** | ('cold exposure'/exp OR 'cold climate'/exp OR 'low temperature':ti,ab,kw OR 'ambient temperature':ti,ab,kw OR 'cold weather':ti,ab,kw OR 'cold spell*':ti,ab,kw OR 'cold wave*':ti,ab,kw) AND ('cardiovascular disease'/exp OR cardiovascular:ti,ab,kw OR 'heart disease*':ti,ab,kw OR coronary:ti,ab,kw OR myocardial:ti,ab,kw OR stroke:ti,ab,kw OR cerebrovascular:ti,ab,kw) AND ('mortality'/exp OR mortalit*:ti,ab,kw OR death*:ti,ab,kw OR fatal*:ti,ab,kw) AND [embase]/lim AND [1900-2026]/py; records published after 30 June 2026 were removed during eligibility assessment. |
| **Web of Science Core Collection** | TS=(("cold exposure" OR "low temperature*" OR "ambient temperature*" OR "cold weather" OR "cold spell*" OR "cold wave*") AND (cardiovascular OR "cardiovascular disease*" OR "heart disease*" OR coronary OR myocardial OR stroke OR cerebrovascular) AND (mortalit* OR death* OR fatal*)) AND PY=(1900-2026); records published after 30 June 2026 were removed during eligibility assessment. |
| **Cochrane Library (Wiley)** | ([mh "Cold Temperature"] OR "cold exposure":ti,ab,kw OR "low temperature*":ti,ab,kw OR "ambient temperature*":ti,ab,kw OR "cold weather":ti,ab,kw OR "cold spell*":ti,ab,kw OR "cold wave*":ti,ab,kw) AND ([mh "Cardiovascular Diseases"] OR cardiovascular:ti,ab,kw OR "cardiovascular disease*":ti,ab,kw OR "heart disease*":ti,ab,kw OR coronary:ti,ab,kw OR myocardial:ti,ab,kw OR stroke:ti,ab,kw OR cerebrovascular:ti,ab,kw) AND ([mh Mortality] OR mortalit*:ti,ab,kw OR death*:ti,ab,kw OR fatal*:ti,ab,kw), with publication date restricted to 30 June 2026 or earlier. |
| **CNKI** | SU=(寒冷 OR 冷暴露 OR 低温 OR 环境温度 OR 寒潮 OR 冷空气) AND SU=(心血管 OR 心血管疾病 OR 冠心病 OR 心肌梗死 OR 脑卒中 OR 脑血管) AND SU=(死亡 OR 死亡率 OR 死亡风险 OR 归因死亡); publication date: database inception to 30 June 2026; literature language: Chinese or English. |

## Supplementary Table S2. SANRA-based self-assessment

The six SANRA items were assessed on the original 0-2 scale. This self-assessment is intended to document reporting features and does not convert the narrative review into a systematic review. Total score: 11/12.

Table 3. SANRA self-assessment of the methodological quality of the narrative review

| **SANRA item** | **Score** | **How the manuscript addresses the item** | **Location** |
| --- | --- | --- | --- |
| 1. Justification of the article's importance | 2 | Explains the global cardiovascular burden of cold exposure and the need to understand regional heterogeneity. | Introduction |
| 2. Statement of concrete aims or formulation of questions | 2 | States the review objective and defines the mechanistic, regional, and determinant-focused synthesis domains. | End of Introduction; Literature Search Strategy and Evidence Selection |
| 3. Description of the literature search | 1 | Reports databases, search date, coverage, languages, eligibility framework, selection responsibility, thematic counts, and full strategies. Original database-level retrieval counts were not preserved. | Literature Search Strategy and Evidence Selection; Supplementary Appendix S1 |
| 4. Referencing | 2 | Key epidemiological, experimental, review, methodological, and contextual claims are supported with Vancouver-style references. | Throughout the manuscript; References |
| 5. Scientific reasoning | 2 | Separates association from causation, distinguishes forms of adaptation, and discusses confounding, exposure heterogeneity, and limitations of cross-regional comparison. | Regional synthesis; Determinants; Discussion |
| 6. Appropriate presentation of endpoint data | 2 | Presents effect measures in their original analytical context and explicitly avoids treating per-degree risks, cold-spell risks, attributable fractions, and excess deaths as equivalent. | Literature Search Strategy and Evidence Selection; Global Regional Heterogeneity; Table 1 |
